# Co-targeting of Cyclooxygenase-2 and FoxM1 is a viable strategy in inducing anticancer effects in colorectal cancer cells

**DOI:** 10.1186/s12943-015-0406-1

**Published:** 2015-07-10

**Authors:** Maqbool Ahmed, Azhar R Hussain, Abdul K. Siraj, Shahab Uddin, Nasser Al-Sanea, Fouad Al-Dayel, Mohammed Al-Assiri, Shaham Beg, Khawla S. Al-Kuraya

**Affiliations:** Human Cancer Genomic Research, Research Center, Riyadh, Saudi Arabia; Department of Surgery, Colorectal unit, Riyadh, Saudi Arabia; Department of Pathology, King Faisal Specialist Hospital and Research Center, Riyadh, Saudi Arabia; Department of Surgery, Security Forces Hospital, Riyadh, Saudi Arabia; Al-Faisal University, Riyadh, Saudi Arabia

**Keywords:** Colorectal cancer, Cox-2, FoxM1, Cell viability, Colony formation, Invasion

## Abstract

**Background:**

Cross-talk between deregulated signaling pathways in cancer cells causes uncontrolled growth and proliferation. These cancers cells become more aggressive and quickly develop resistance to therapy. Therefore targeting of these deregulated pathways simultaneously can result in efficient cell death of cancer cells. In this study we investigated co-expression of Cox-2 and FoxM1 in a cohort of colorectal carcinoma (CRC) samples and also examined whether inhibition of Cox-2 and FoxM1 simultaneously can lead to inhibition of cell viability and induction of apoptosis in colorectal cancer cell lines and *in vivo* xenografts.

**Methods:**

Protein expression of Cox-2 and FoxM1 was determined in a large cohort of 770 clinical CRC samples in a tissue micro-array format by immunohistochemistry. Cell death was measured using live dead assay. Apoptosis was measured by annexin V/PI dual staining. Immunoblotting was performed to examine the expression of proteins. Calcusyn software was utilized to estimate the synergistic doses using chou and Talalay method.

**Results:**

Co-expression of Cox-2 and FoxM1 was detected in 33.3 % (232/697) of CRC’s and associated with an aggressive phenotype characterized by younger age (*p* = 0.0191), high proliferative index marker; Ki-67 (*p* = 0.004) and MMP-9 (*p* = 0.0116) as well as activation of AKT (*p* = 0.0214). *In vitro,* inhibition of FoxM1 and Cox-2 with pharmacological inhibitors; Thiostrepton and NS398 resulted in efficient down-regulation of FoxM1 and Cox-2 expression along with in-activation of AKT and inhibition of colony formation, invasion and migratory capability of CRC cells. In addition, there was also inhibition of cell viability and induction of apoptosis via the mitochondrial apoptotic pathway in CRC cell lines. Finally, treatment of CRC xenograft tumors in nude mice with combination of Cox-2 and FoxM1 inhibitors inhibited tumor growth significantly via down-regulation of Cox-2 and FoxM1 expression.

**Conclusions:**

These findings demonstrate that co-expression of Cox-2 and FoxM1 might play a critical role in the pathogenesis of CRC. Therefore, targeting of these pathways simultaneously with sub toxic doses of pharmacological inhibitors can be a potential therapeutic approach for the treatment of this subset of CRC.

**Electronic supplementary material:**

The online version of this article (doi:10.1186/s12943-015-0406-1) contains supplementary material, which is available to authorized users.

## Background

Despite increased awareness in the general population regarding colorectal cancer (CRC), it still remains a major cause of mortality and morbidity worldwide [1]. This increase has been attributed to a combination of environmental and genetic factors in the general population [2, 3]. Even though CRC is very well studied along with established diagnostic markers, most of CRC cancers present at late stages of disease and therefore have a poor prognosis [4]. In addition, recurrence and metastasis of CRC also carry a very high mortality rate [5]. Therefore, there is a need for improvement in the diagnosis of CRC as well as identification of newer therapeutic targets that can be specifically drugged to improve the management of these cancers.

An important key survival molecule that is currently being investigated as a molecular marker and a potential therapeutic target is cyclooxygenase-2 (Cox-2) in various cancers. The main function of Cox is to synthesize prostaglandins from arachidonic acid [6]. There are two isoforms of Cox; Cox-1 that is found to be expressed in normal cells [7] and Cox-2 that is preferentially expressed in cancer cells [7] and its expression is enhanced by pro-inflammatory cytokines and carcinogens [8, 9]. Cox-2 has been found to be over-expressed by us and others in a variety of cancer including breast, ovary, colorectal, thyroid and lung [10–14]. Prophylactic use of Cox-2 inhibitors such as aspirin has been shown to decrease the incidence of certain cancers [15–18].

Forkhead box protein M1 (FoxM1) is a member of FoxM family that consists of more than 50 proteins that are characterized by a conserved 100 amino acid DNA binding domain [19, 20]. FoxM1 has also been known to regulate the transcriptional activity of number of genes including cyclin B, cyclin A and Aurora B kinase, which are very important for cell cycle progression and mitotic entry [21–23]. Loss of FoxM1 expression has also been reported to generate mitotic spindle defects leading to mitotic catastrophe [21, 24, 25]. FoxM1 signaling has been implicated to be associated with carcinogenesis of tumor development in CRC as well as other solid tumors [22, 26–33].

A number of dysregulated survival pathways have the ability to cross-talk with each other to increase aggressiveness of various cancers [34, 35]. These cross-talks allow the cancer cell to avoid different *in vivo* and *in vitro* threats thereby allowing un-supervised growth and proliferation and the cancers cells become more aggressive and quickly develop resistance to therapy [35]. Inhibiting one pathway may not be enough to elicit a complete response because of the cross-talk with other pathways thereby eliciting a feedback response to reactivate the targeted pathway [36]. Targeting multiple pathways also helps in decreasing drug-induced toxicity by using sub-toxic doses in combination.

There have been many studies performed to investigate the role of Cox-2 and FoxM1 in tumorigenesis independently however there are only few studies where these molecules are studied together [37]. Therefore, in this study, we first investigated co-expression of Cox-2 and FoxM1 in CRC clinical samples followed by determining whether targeting of co-expression of FoM1 and Cox-2 can generate efficient anticancer effects in CRC cells both *in vitro* as well as *in vivo* models.

## Results

### Evaluation of molecular expression of Cox-2 and FoxM1 in CRC tissues

Immunohistochemical analysis of Cox-2 expression was interpretable in 726 CRC spots and the incidence of Cox-2 over-expression was found to be 60.6 % (440/726). FoxM1 expression was interpretable in 719 CRC spots and the incidence of FoxM1 over-expression was found to be 50.3 % (362/719). Cox-2 was seen predominantly in cytoplasmic compartment and FoxM1 expression was seen predominantly in the nuclear compartment. Co-expression of Cox-2 and FoxM1 was seen in 33.3 % (232/697) of cases and were significantly associated with each other (*p* = 0.0115). Co-expression of Cox-2 and FoxM1 were found to be significantly associated with clinical parameters such as younger age (*p* = 0.0191) and mucinous histology (*p* = 0.0174) but were not associated with sex, or American Joint Committee on Cancer (AJCC) stage. Molecular association of this co-expression was seen with proliferative marker Ki-67 (*p* = 0.0004), p-AKT (*p* = 0.0214) and MMP-9 (*p* = 0.0116) (Table 1). No survival difference was seen between patients showing Cox-2 and FoxM1 co-expression and those with normal or reduced expression (*p* = 0.4796) (Table 1 and Additional file 1: Figure S1).

**Table 1:** Correlation of Cox-2 & Fox-M1 co-expression with clinico-pathological parameters in colorectal carcinoma^a^

	Total		Both high		Any-1-low		*p* value
	Number	Percent	Number	Percent	Number	Percent	
Total number of cases	697		232	33.3	465	66.7	
Age							
≤50 years	229	32.9	90	39.3	139	60.7	0.0191
>50 years	468	67.1	142	30.3	326	69.7	
Sex							
Male	358	51.4	116	32.4	242	67.6	0.6111
Female	339	48.6	116	34.2	223	65.8	
Tumour Site							
Left colon	548	82.3	176	32.1	372	67.9	0.5821
Right colon	118	17.7	41	34.8	77	65.2	
Histological Type							
Adenocarcinoma	628	90.1	200	31.9	428	68.1	0.0174
Mucinous Carcinoma	69	9.9	32	46.4	37	53.6	
Tumour Stage							
I	80	12.2	25	31.3	55	68.7	0.4160
II	233	35.4	75	32.2	158	67.8	
III	266	40.4	98	36.8	168	63.2	
IV	79	12.0	22	27.8	57	72.2	
Differentiation							
Well	66	9.5	21	31.8	45	68.2	0.3709
Moderate	549	78.8	178	32.4	371	67.6	
Poor	82	11.8	33	40.2	49	59.8	
Ki-67							
High	590	87.7	209	35.4	381	64.6	0.0004
Low	83	12.3	14	16.9	69	83.1	
p-AKT							
High	454	72.3	169	37.2	285	62.8	0.0214
Low	174	27.7	48	27.6	126	72.4	
MMP-9							
High	342	52.2	129	37.7	213	62.3	0.0116
Low	313	47.8	89	28.4	224	71.6	
Survival							
OS 5 Years				73.6		68.5	0.4796

^a^Data were not available for (Tumor Site NA = 31), (Tumor Stage NA = 39), (Ki-67 NA = 24), (p-AKT NA = 69) and (MMP-9 NA = 42)

### Inhibition of Cox-2 and FoxM1 causes inhibition of cell viability in CRC cell lines

**Fig. 1:**
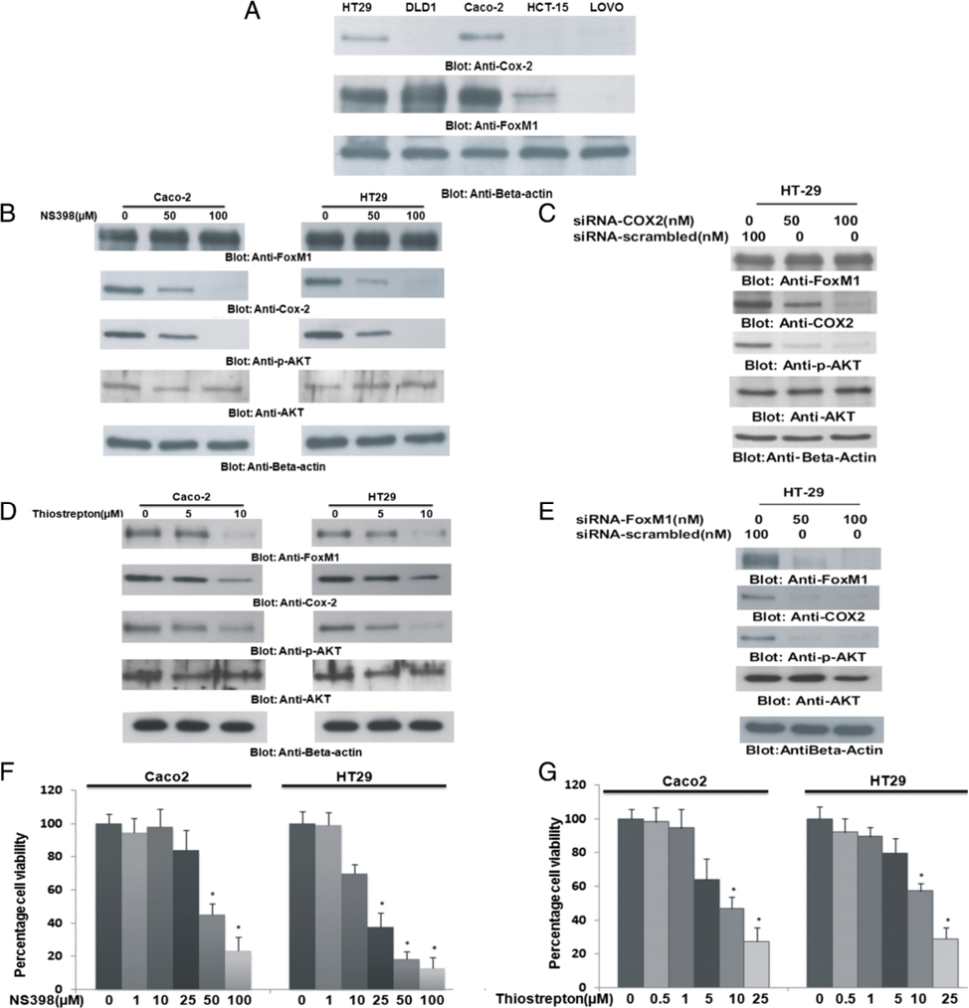
NS398 and Thiostrepton inhibits cell viability in CRC cell lines (**a**) HT29, DLD1, Caco-2, HCT-15 and LOVO cells were lysed and immuno-blotted with Cox-2, FoxM1 and Beta-actin antibodies (**b**) Caco-2 and HT29 cell lines were treated with 50 and 100 μM NS398 for 48 h. Proteins were lysed and immunoblotted with antibodies against FoxM1, Cox-2, p-AKT, total AKT and Beta-actin. **c** HT29 cells were either transfected with 50 and 100nM siRNA, specific against Cox-2 or scrambled siRNA for 48 h. Cells were lysed and equal amounts of proteins were immuno-blotted with antibodies against FoxM1, Cox-2, p-AKT, total AKT and Beta-actin. **d** Caco-2 and HT29 cell lines were treated with 5 and 10 μM Thiostrepton for 48 h. Proteins were lysed and immunoblotted with antibodies against FoxM1, Cox-2, p-AKT, total AKT and Beta-actin. **e** HT29 cells were either transfected with 50 and 100nM siRNA, specific against FoxM1 or scrambled siRNA for 48 h. Cells were lysed and equal amounts of proteins were immuno-blotted with antibodies against FoxM1, Cox-2, p-AKT, total AKT and beta-actin. **f** Caco-2 and HT29 cell lines were incubated with 0-100 μM NS398 for 48 h. Cell viability was measured by MTT assays as described in Materials and Methods. The graph displays the mean +/- SD (standard deviation) of three independent experiments, * *p* < 0.05, statistically significant (Students *t*-test). **g** Caco-2 and HT29 cell lines were incubated with 0-25 μM Thiostrepton for 48 h. Cell viability was measured by MTT assays as described in Materials and Methods. The graph displays the mean +/- SD (standard deviation) of three independent experiments, * *p* < 0.05, statistically significant (Students *t*-test)

*In vitro,* we initially sought to determine expression of Cox-2 and FoxM1 in a panel of CRC cell lines by immuno-blotting. We found that out of five CRC cell lines, only HT29 and Caco-2 had constitutive co-expression of Cox-2 and FoxM1 (Fig. 1a) therefore we selected these two cell lines in our study. We next determined the effect of Cox-2 inhibitor NS398 and FoxM1 inhibitor Thiostrepton [38] that has also been shown to possess proteasomal inhibition activity [39] on the expression of these proteins. At first, Caco-2 and HT29 cells were treated with 50 and 100 μM NS398 for 48 h. NS398 treatment failed to down-regulate the expression of FoxM1 in both the cell lines, even though, expression of Cox-2 was down-regulated and there was inactivation of AKT (Fig. 1b). This data was further confirmed by transfecting HT29 cells with specific siRNA targeted against Cox-2. As shown in Fig. 1c, similar results were obtained where there was no effect on the expression of FoxM1 in CRC cell lines while the expression of Cox-2 decreased and there was in-activation of AKT following transfection with siRNA targeting Cox-2. In a separate experiment, CRC cell lines were treated with 5 and 10 μM Thiostrepton for 48 h and immunoblotted with FoxM1, Cox-2, p-AKT and total AKT antibodies. The doses of Thiostrepton used have been previously shown to down-regulate expression of FoxM1 in other tumor cell lines without any off target effect or toxicity to normal peripheral blood mononuclear cells (PBMNC) [40, 41]. As shown in Fig. 1d, Thiostrepton treatment down-regulated expression of FoxM1 and Cox-2 and caused dephosphorylation of AKT at 10 μM in both the cell lines. Similar results were obtained when CRC cell lines were transfected with siRNA targeted against FoxM1 for 48 h and immunoblotted with antibodies against FoxM1, Cox-2, p-AKT and total AKT (Fig.1e). These data suggest that FoxM1 is expressing upstream of Cox-2 and there is a link between FoxM1 and Cox-2 in CRC cells. Finally, we sought to determine whether treatment of CRC cell lines with Cox-2 and FoxM1 inhibitors leads to inhibition of cell viability. Caco-2 and HT29 were cultured in the presence of 1, 10, 25, 50 and 100 μM NS398 for 48 h and cell viability was assayed using MTT assay. As shown in Fig. 1f, there was a dose dependent inhibition of cell viability in both the cell lines that reached significance at 50 μM for Caco-2 and 25 μM for HT29 respectively (p < 0.05). Interestingly, this response was not seen in Cox-2 deficient DLD1 and LOVO cells up to doses of 100 μM NS398 (Additional file 2: Figure S2 and Additional file 3: Figure S3). Similarly, the two cell lines were cultured in the presence of 0.5, 1, 5, 10 and 25 μM Thiostrepton for 48 h. Data revealed that there was also a dose dependent response to Thiostrepton treatment that reached statistical significance at 10 μM for HT29 and Caco-2 cell lines respectively (Fig. 1b). In addition, there was partial response in FoxM1 negative cell line; LOVO as shown in Additional file 3: Figure S3. These data suggest that targeting Cox-2 and FoxM1 using specific inhibitors led to inhibition of cell viability in CRC cells.

### Synergistic activity of thiostrepton and NS398 in CRC cell lines

As our data showed FoxM1 and Cox-2 co-expression was present in CRC, we hypothesized that targeting of FoxM1 and Cox-2 expression together can lead to efficient cytotoxic effects in CRC cells. Therefore we sought to determine whether co-treatment of CRC cell lines with Thiostrepton, and NS398 at sub-toxic doses, can potentiate anticancer effects in CRC cells.

**Fig. 2:**
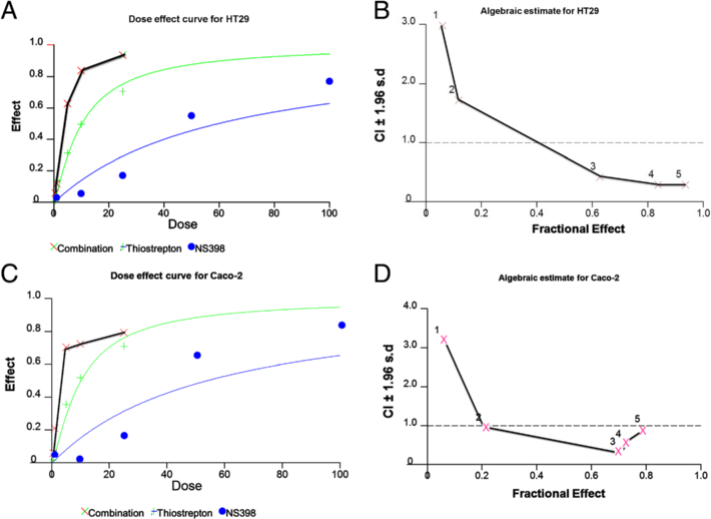
Calculation of optimal doses required for co-treatment of CRC cells by Thiostrepton and NS398. HT29 and Caco-2 cells were treated with 0.5, 1.0, 5.0, 10 and 25 μM Thiostrepton or 1.0, 10, 25, 50 and 100 μM NS398 alone or in different combinations to calculate the Synergistic apoptotic response of Thiostrepton and NS398 for 48 h and dose effect (**a** and **c**) and Fractional effect (**b** and **d**) graphs were generated using Calcusyn software. Apoptotic response analysis was done as mean ± SD values normalized to control. Combination indices were calculated using Chou and Talalay methodology

**Fig. 3:**
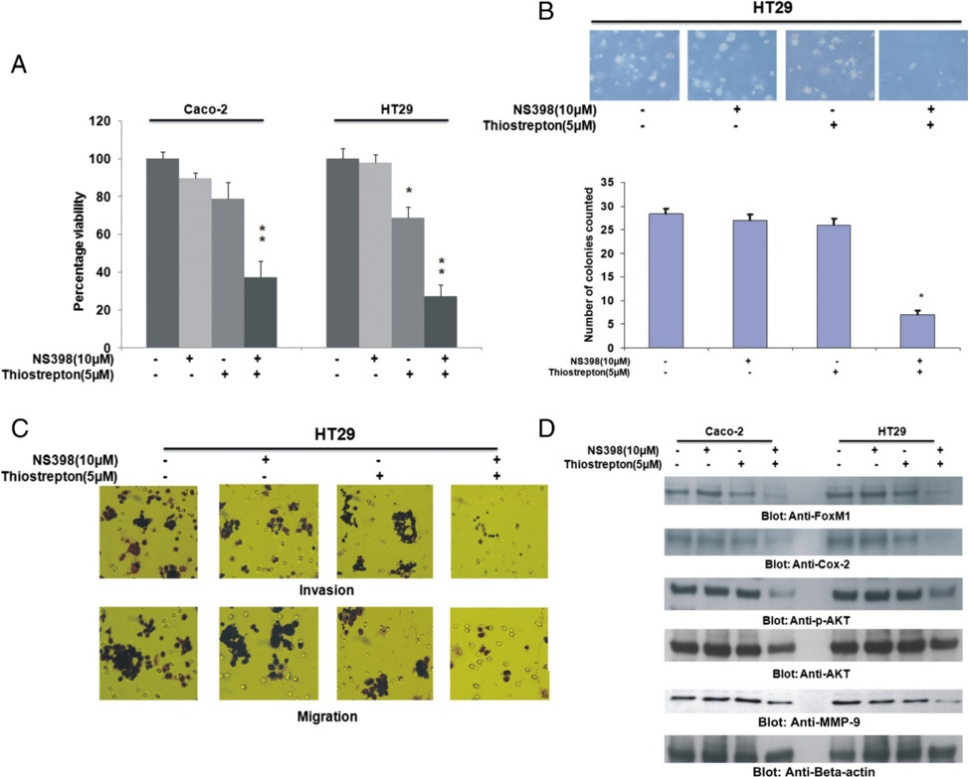
Combination of NS398 and Thiostrepton at sub-optimal doses inhibits cell viability and decreases cell invasion/migration capability of CRC cells. **a** Caco-2 and HT29 cell lines were incubated with either 10 μM NS398 or 5 μM Thiostrepton alone or in combination for 48 h. Cell viability was measured by MTT assays as described in Materials and Methods. The graph displays the mean +/- SD (standard deviation) of three independent experiments, * *p* < 0.05, statistically significant (Students *t*-test). **b** Colony formation assays were performed as described in Materials and Methods. HT29 cells were treated with either 10 μM NS398 or 5 μM Thiostrepton alone or in combination for 48 h. Subsequently, cells were plated on Soft agar plates for 4 weeks. After 4 weeks, plates were stained and manually counted. Bar graph denotes number of colonies counted manually. **c** HT29 cells were treated with 10 μM NS398 and 5 μM Thiostrepton alone or in combination for 48 h. Following treatment, Invasion-Migration assay were performed as described in the material and methods section. **d** Caco-2 and HT29 cell lines were treated with 10 μM NS398 and 5 μM Thiostrepton either alone or in combination for 48 h. Proteins were lysed and immunoblotted with antibodies against FoxM1, Cox-2, p-AKT, total AKT, MMP-9 and Beta-actin

We conducted multiple experiments to determine the optimal doses that could be used in combination to inhibit cell viability, migration and colony formation and induce apoptosis in CRC cell lines. Using Chou and Talalay method [42], we found that 5 μM Thiostrepton and 10uM NS398 in combination exerted maximum synergistic apoptotic response in HT29 and Caco-2 cells with combination index of 0.286 and 0.332 respectively (Fig. 2, Additional file 4: Table S1, Additional file 5: Table S2). Therefore, we first treated CRC cells with sub-optimal doses of Thiostrepton (5 μM) and NS398 (10 μM) for various time points and found that the optimal synergistic response was detected at 48 h following treatment (Additional file 6: Figure S4). Therefore, we treated Caco-2 and HT29 cells with combination of NS398 and Thiostrepton for 48 h and assessed the cell viability by MTT assay. As shown in Fig. 3a, neither Thiostrepton nor NS398 alone could inhibit cell viability, however, co-treatment with Thiostrepton and NS398 led to significant inhibition of cell viability in CRC cells (p < 0.01). Next, we determined whether combination of Thiostrepton and NS398 could inhibit colony formation in CRC cells. As shown in Fig. 3b, there was significant inhibition in colony formation in HT29 cells as compared to treatment alone. Combination of Thiostrepton and NS398 also inhibited cell invasion and migration in HT29 cells as compared to treatment alone (Fig. 3c). We finally investigated whether co-treatment of CRC cells with sub-optimal doses of Thiostrepton and NS398 could inhibit expression of FoxM1, Cox-2 and MMP-9 by immunoblotting. As shown in Fig. 3d, combination treatment of Thiostrepton and NS398 successfully down-regulated expression of FoxM1, Cox-2 and MMP-9 and inactivated p-AKT without disrupting the expression of total AKT in Caco-2 and HT29 cell lines. These data indicate that co-treatment with Thiostrepton and NS398 synergistically inhibits cell viability and migratory properties of CRC cells. Finally, Cox-2 deficient cell line; DLD1 cells and Cox-2 and FoxM1 deficient cell line; LOVO did not show any synergistic response when treated with combination of 5 μM Thiostrepton and 10 μM NS398 and the cell inhibition detected were due to Thiostrepton treatment alone (Additional file 7: Table S3 and Additional file 8: Table S4) confirming specificity of NS398 and Thiostrepton treatment against Cox-2 and FoxM1expression.

### Co-treatment of CRC cells with thiostrepton and NS398 induces apoptosis via mitochondrial apoptotic pathway

**Fig. 4:**
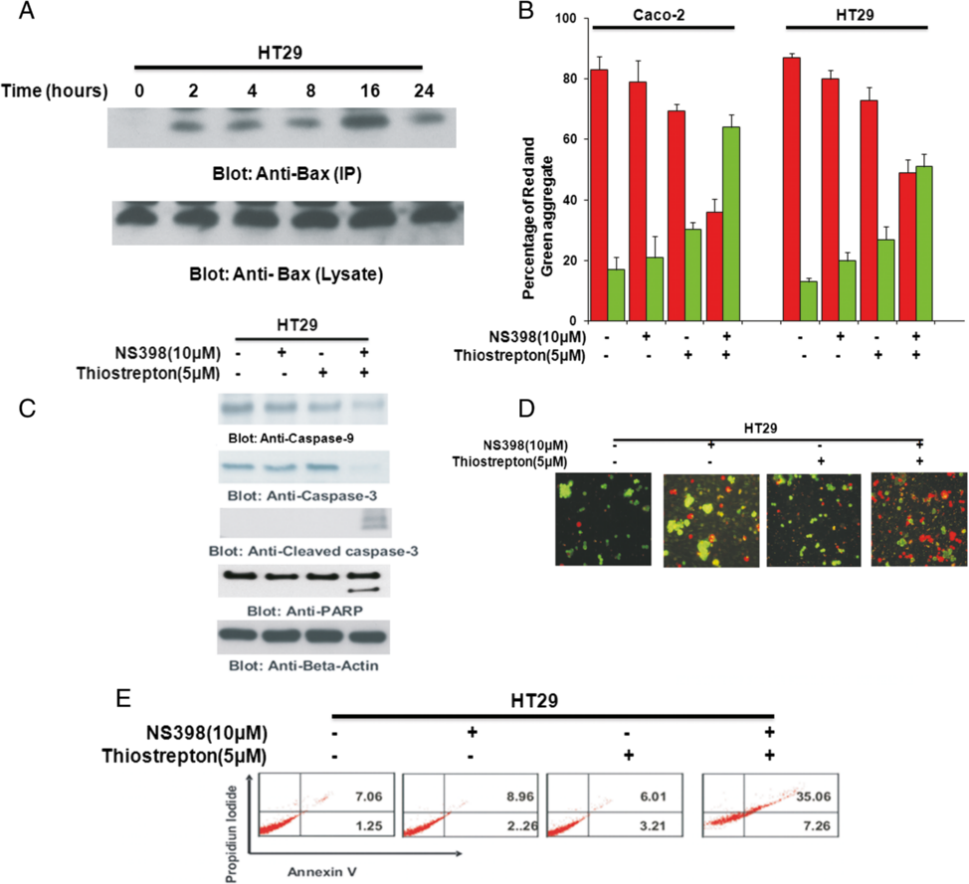
Combination of NS398 and Thiostrepton at sub-optimal doses induces caspase-dependent apoptosis via the mitochondrial pathway in CRC cells. **a** HT29 cells were treated with combination of 10 μM NS398 and 5 μM Thiostrepton for indicated time periods. Cells were lysed in 1 % Chaps lysis buffer and subjected to immuno-precipitation with anti-Bax 6A7 monoclonal antibody and probed with specific polyclonal anti-Bax antibody for detection of conformationally changed Bax protein. In addition, the total cell lysates were applied directly to SDS–PAGE, transferred to immobilon membrane and immuno-blotted with specific anti-Bax polyclonal antibody. **b** Caco-2 and HT29 cells were treated with 10 μM NS398 and 5 μM Thiostrepton either alone or in combination for 48 h. Live cells with intact mitochondrial membrane potential (red bars) and dead cells with lost mitochondrial membrane potential (green bars) was measured by JC-1 staining and analyzed by flow cytometry as described in Materials and Methods. The graph displays the mean +/- SD (standard deviation) of three independent experiments. **c** HT29 cells were treated with 10 μM NS398 and 5 μM Thiostrepton alone or in combination for 48 h. Cells were lysed and equal amounts of proteins were immunoblotted with antibodies against caspase-9, caspase-3, cleaved caspase-3, PARP, and Beta-actin. **d** Caco2 and HT29 cells were treated with 10 μM NS398 and 5 μM Thiostrepton either alone or in combination for 48 h and cell death was analyzed by Live/Dead Assay. **e** HT29 cells were treated with 10 μM NS398 and 5 μM Thiostrepton alone or in combination for 48 h. Thereafter, the cells were washed, and stained with annexin V/propidium iodide, and analyzed by flow cytometry as described in Materials and methods

For efficient apoptosis to occur, the mitochondrial apoptotic pathway needs to be activated. Bax, a pro-apoptotic BH3 domain only protein of the Bcl-2 family [43] is the first protein that undergoes conformational changes for the mitochondrial apoptotic pathway to be activated [44]. To investigate the effect of co-treatment with Thiostrepton and NS398 on activation of the mitochondrial pathway, we treated HT29 cells for various time periods and examined the conformational changes in Bax protein by immuno-precipitation. As shown in Fig. 4a, conformationally changed Bax was detected after 2 h, peaked within 16 h and then decreased at 24 h of treatment with 5 μM Thiostrepton and 10 μM NS398. Once Bax is conformationally changed, it causes changes in the mitochondrial membrane potential. To investigate this, we treated Caco-2 and HT29 cells with either Thiostrepton or NS398 alone or in combination for 48 h and examined the change in mitochondrial membrane potential by flow cytometry. Neither Thiostrepton nor NS398 could affect the mitochondrial membrane potential alone however when combination of both inhibitors were used together, there was an increase in cells undergoing mitochondrial membrane damage as depicted by green bars (Fig. 4b). Once there are changes in the mitochondrial membrane potential, there is release of cytochrome into the cytosole (Data not shown) leading to activation and cleavage of downstream caspases. To determine this, we treated HT29 cells with NS398 (10 μM), Thiostrepton (5 μM) and a combination of the two for 48 h and examined cleavage of caspase-9, caspase-3 and PARP by immunoblotting. Caspase-9, -3 and PARP were cleaved in cells that were treated with combination of Thiostrepton and NS398 (Fig. 4c). Once the downstream caspases are activated and cleaved, it leads to cell death via apoptosis. To confirm this, we visualized cells under an Olympus fluorescent microscope using a longpass filter after 48 h treatment with NS398, Thiostrepton or a combination of two inhibitors after staining them with 50 μM calcein AM and 8 μM ethidium homodimer. As shown in Fig. 4d, there were more green stained cells depicting live cells in samples treated with Thiostrepton or NS398 alone. The cells stained red representing dead cells in sample that was treated with combination of Thiostrepton and NS398. Apoptosis was further confirmed by annexinV/PI dual staining that was investigated by flow cytometry. These set of data confirm that combination of Thiostrepton and NS398 treatment induces apoptosis in CRC cells via activation of mitochondrial apoptotic pathway.

### Inhibition HT29 xenografts by combinational treatment of thiostrepton and NS398 in nude mice

**Fig. 5:**
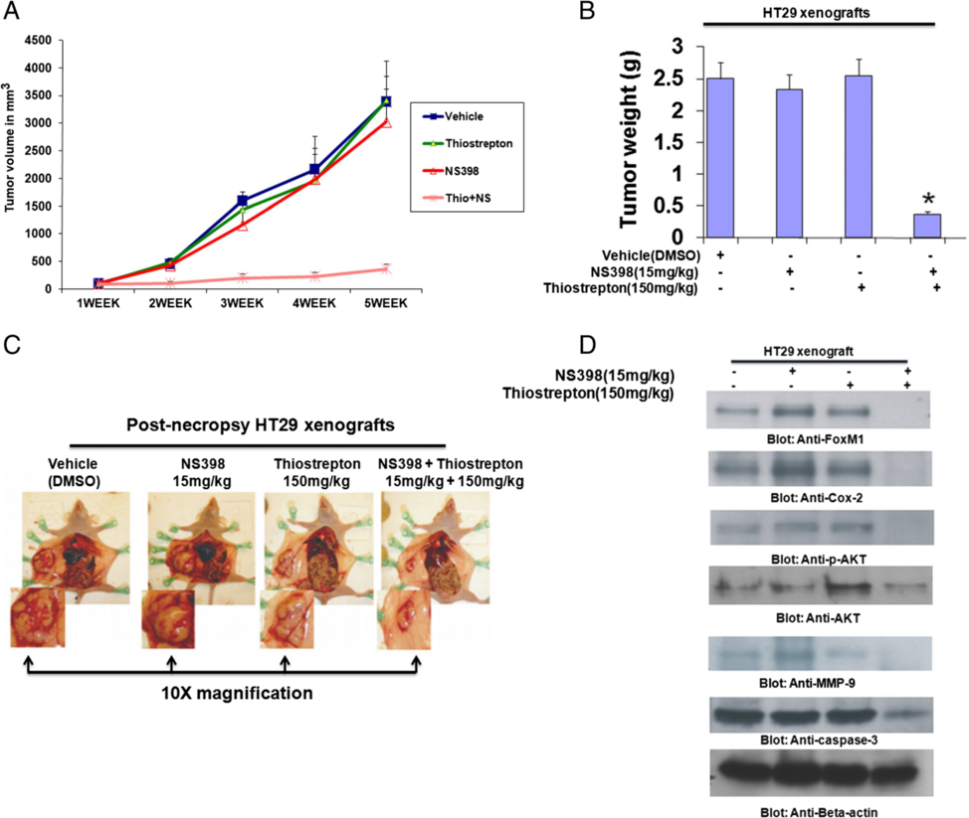
Combination of NS398 and Thiostrepton at sub-optimal inhibits growth of HT29 xenograft and down-regulates expression of Cox-2, FoxM1 and p-AKT. Nude mice at 6 weeks of age were injected with 10 million HT29 cells. **a** The volume of each tumor was measured every week. The average (*n* = 6) tumor volumes in vehicle-treated mice () and mice treated with indicated doses of NS398 (15 mg/kg ), Thiostrepton (150 mg/kg ) and a combination of NS398 and Thiostrepton (NS398 15 mg/kg + Thiostrepton 150 mg/kg; ) were plotted. The results are expressed as mean ± SD (*n* = 6). *P <0.001 compared with vehicle-treated mice. **b** After 5 weeks of treatment, mice were sacrificed and tumor weights were measured. The results are expressed as mean ± SD. *P < 0.001 compared with vehicle-treated mice by Student *t* test. **c** Representative tumor images of vehicle-treated mice and mice treated with NS398, Thiostrepton and combination of both agents after necropsy. **d** Whole cell homogenates from mice treated with vehicle, 15 mg/kg NS398, 150 mg/kg Thiostrepton and a combination of 15 mg/kg NS398, + 150 mg/kg Thiostrepton were immuno -blotted with FoxM1, Cox-2, p-AKT, total AKT, MMP-9, caspase-3, and Beta-actin antibodies

The synergistic effect of the combination of Thiostrepton and NS398 *in vitro* suggested that this combination would likely to be effective in tumor xenografts *in vivo*. Therefore, we sought to determine whether co-treatment of Thiostrepton with NS398 potentiated the inhibition of CRC cell line generated xenograft tumor in nude mice. For xenograft study, mice were inoculated subcutaneously into the right abdominal quadrant with10 million HT29 cells in 200 μl PBS. After 1 week of inoculation, mice were randomly assigned into four groups: The first group received DMSO as control vehicle while the other three groups received NS398 (15 mg/kg), Thiostrepton (150 mg/kg) and combination of 15 mg/kg NS398 and 150 mg/kg Thiostrepton, injected twice weekly, intraperitoneally respectively. After 5 weeks treatment, mice were sacrificed and tumors were collected. As shown in Fig. 5a, there was significant regression of tumor volume at the end of second week in the group of animal treated with Thiostrepton and NS398. Neither Thiostrepton nor NS398 alone resulted in significant inhibition of xenograft tumors. A significant reduction in tumor weight (Fig. 5b) was also observed in mice treated with Thiostrepton and NS398 (p < 001). We also visualized images of the tumor, post-necropsy and found that there was significant shrinkage in the size of the tumor following treatment with combination of Thiostrepton and NS398 as compared to treatment alone (Fig. 5c). Finally, we analyzed the status FoxM1, Cox-2, p-AKT, total AKT and caspase-3 in HT29 xenograft at the end of study. As shown in Fig. 5d, the level of FoxM1, Cox-2, MMP-9 and caspase-3 were remarkably deceased along with in-activation of AKT in tumors of mice co-treated with Thiostrepton and NS398, compared to vehicle, Thiostrepton and NS398 alone treatment. Our data indicates that co-treatment with Thiostrepton and NS398 augmented antitumor effects in HT29 cell xenografts in Nude mice.

## Discussion

In this study, we have investigated the role of Cox-2 and FoxM1 co-expression in a large cohort of 770 Middle Eastern CRC in a tissue microarray format to determine the protein expression by immunohistochemistry. It has been previously shown that Cox-2 and FoxM1 expression are associated with a poor prognosis in CRC [45, 46]. In this study, we found a significant association between FoxM1 and Cox-2 over-expression in Middle Eastern CRC samples (*p* = 0.0115). Co-expression of FoxM1 and Cox-2 was also found to be associated with an aggressive phenotype that was characterized by younger age (*p* = 0.0191), proliferative marker Ki-67 (0.0004), MMP-9 (*p* = 0.0116) and activation of AKT (*p* = 0.0214). These data reiterates the point that targeting these two molecules simultaneously using small molecular inhibitors may be more beneficial for the management of this aggressive phenotype of CRC when compared to treatment with single agent.

Cross talk between survival pathways is slowly emerging as one of the leading causes of drug resistance to small molecular inhibitors for the treatment of cancer. After an initial response to the treatment, resistance to therapy quickly develops due to re-activation of the target molecule either by up-stream pro-survival pathway molecules or negative feed-back mechanism by down-stream molecules. To counteract this resistance, recent studies have shown targeting multiple pro-survival molecules of different survival pathways that are associated with each other with combination of specific inhibitors simultaneously is more beneficial than treatment with single agent alone [47, 48]. Combination treatment is also beneficial because the dose of each drug is considerably decreased when used in combination thereby reducing the chances of toxicity to normal cells. Therefore, the role of single agent treatment with molecular inhibitors is diminishing and targeting various cancers with multiple inhibitors is on the rise.

A strong association between expression of FoxM1 and Cox-2 with MMP-9 expression has been reported in a number of cancers [31, 49, 50]. Previously, it has also been reported in lung cancer that transcriptional depletion of FoxM1 expression can cause reduced Cox-2 expression and on the other hand, induced over-expression of FoxM1 protein can increase Cox-2 promoter activity [37]. These findings are in conco rdance with our data where pharmacological and transcriptional inhibition of FoxM1 expression down-regulates the expression of Cox-2. Furthermore, our data also demonstrates that depletion of Cox-2 expression does not affect FoxM1 expression suggesting that FoxM1 is functional upstream of Cox-2. Combined targeting of Cox-2 and FoxM1 with pharmacological inhibitors also depletes colony formation as well as invasive and migratory capabilities of CRC cells *in vitro* via down-regulation of MMP-9 thereby indicating the utility of combined targeting of these molecules for inhibition of metastasis in CRC cells.

Combined targeting of Cox-2 and FoxM1 not only inhibits the invasive and migratory capability of CRC cells, they also cause inhibition of cell viability and induction of apoptosis. This effect occurs via inactivation of an important survival molecule; p-AKT, that plays an important role in the survival of cancer cells and is found to be constitutively activated in various cancers [51]. Our data showed that dephosphorylation of AKT led to activation of the mitochondrial apoptotic pathway initiated by Bax conformational changes and translocation to the mitochondrial membrane, thereby leading to changes in the mitochondrial membrane potential and finally activation and cleavage of caspases. Once caspases are activated, there is cleavage of PARP; an essential enzyme that is required for repairing single stranded breaks in DNA [49] and is a hallmark of cells undergoing apoptosis. Our *in vivo* studies further validate our hypothesis that co-treatment of mice bearing palpable CRC xenograft with Thiostrepton and NS398 leads to regression of tumor growth via down-regulation of FoxM1, Cox-2, MMP-9, inactivation of AKT and cleavage of caspase-3 which is consistent with our *in vitro* findings.

## Conclusions

Altogether, we found that 33.3 % of CRC clinical samples co-express Cox-2 and FoxM1 and this sub-group is associated with an aggressive phenotype. Therefore, our data highlights the importance of co-targeting of deregulated survival pathways (Cox-2 and FoxM1) in CRC cells can lead to anticancer effects. Our data showed that combination treatment of CRC cells with sub-optimal doses of Thiostrepton and NS398 caused functional inhibition of Cox-2 and FoxM1 simultaneously. Even though, Thiostrepton and NS398 have been previously shown to be effective in suppressing growth and inducing apoptosis in CRC cells at higher concentrations [31, 52], this study emphasizes the importance of targeting multiple survival molecules with sub-optimal doses of Thiostrepton and NS398 to successfully inhibit cell growth, invasion, migration and induce apoptosis in CRC. Further studies are warranted to further investigate the utility of combination treatment with Thiostrepton and NS398 for the treatment of CRC in clinical settings.

## Material and methods

### Patient selection and tissue microarray construction

Seven hundred and seventy patients with CRC diagnosed between 1990 and 2011 were selected from King Faisal Specialist Hospital and Research Centre. Clinical and histopathological data were available for all these patients. Colorectal Unit, Department of Surgery, provided long-term follow-up data. Patients with colon cancer underwent surgical colonic resection and rectal cancer underwent anterior resection or abdominoperineal resection. Majority of node positive colon cancers received 5-fluorouracil based adjuvant chemotherapy. A vast majority of the rectal cancers received radiotherapy alone or chemo-radiotherapy prior to surgery followed by adjuvant chemotherapy after surgery. Tissue microarrays were constructed from formalin-fixed, paraffin-embedded colorectal carcinoma specimens as described previously [53]. Institutional Review Board (IRB) of the King Faisal Specialist Hospital & Research Centre approved the study (NSTIP 10-BIO-959-20 and RAC 2140 005).

### Immunohistochemistry (IHC)

TMA slides were processed and stained manually as described previously [54]. Sections were deparaffinized in xylene and rehydrated through graded alcohol to water. Antigen retrieval was done in a Pascal Pressure cooker at 120 °C for 8 min using the Dako Retrieval solution, pH 6 (S2369; Dako Cytomation, Copenhagen, Denmark). Endogenous peroxidase activity was blocked by incubating the slides in 3 % H_2_O_2_ in water for 30 min at room temperature. Sections were incubated in 1 % BSA for 30 min then wiped off and dilution of Cox-2 and FoxM1 was applied on the slides and incubated overnight at room temperature. Subsequently sections were incubated with Envision + secondary antibody for 1 h at room temperature and visualization was done using the liquid DAB + substrate chromogen system. Only fresh cut slides were stained simultaneously to minimize the influence of slide ageing and maximize repeatability and reproducibility of the experiment. Details of primary antibodies used, dilutions, cut-off and incidences of positive cases are listed in Additional file 9: Table S5. H-score was used for categorizing the expression of Cox-2 and FoxM1. Each TMA spot was assigned an intensity score from 0 to 3 (I0, I1–I3) and proportion of the tumor staining for that intensity was recorded in 5 % increments from 0 to 100 (P0, P1–P3). A final H score (range, 0–300) was obtained by adding the sum of scores obtained for each intensity I and proportion of area stained. X-tile plots were constructed for assessment of biomarker and optimization of cutoff points based on outcome, as described previously [55]. The CRCs were stratified into two groups based on X-tile plots: one with complete absence or reduced staining and the other with overexpression. For Ki-67 cut-off of ≥50 % nuclear staining was used and for p-AKT intensity score 2+/3+ was considered as positive.

### Statistical analysis

Contingency table analysis and *χ*^2^ tests were used to study relationship between clinicopathological variables and gene amplification. The limit of significance for all analyses was defined as a *P* value of 0.05; two-sided tests were used in all calculations. The JMP 10.0 software package (SAS Institute, Cary, NC) was used for data analyses.

### Reagents and antibodies

Thiostrepton (FoxM1 selective inhibitor) [56] was purchased from Tocris Cookson Inc (Ellisville, MO). NS398 (COX-2 inhibitor) was purchased from Caymen chemical company, (Ann Arbor, MI). Antibodies against cleaved caspase-3, Cox-2, AKT and p-AKT antibodies were purchased from Cell Signaling Technologies (Beverly, MA). FoxM1, Bax, Beta-actin, caspase-3 and poly (ADP) ribose polymerase (PARP) antibodies were purchased from Santa Cruz Biotechnology, Inc. (Santa Cruz, CA). MMP-9 antibodies were purchased from Anespec, (San Jose, CA). Annexin V/PI kit was purchased from Molecular Probes (Eugene, OR, USA).

### Cell culture

HT29, DLD1, LOVO, HCT-15 and Caco-2 were obtained from Deutsche Sammlung von Mikroorganismen und Zellkulturen (DSMZ), Braunschweig, Germany. All cell lines were tested for immunological markers and cytogenetics. The cell lines were also fingerprinted and species was confirmed by IEF of AST, MDH and NP. Cells were cultured in RPMI 1640 medium supplemented with 10 % (vol/vol) fetal bovine serum, 100 U/ml Penicillin and 100 U/ml Streptomycin at 37 °C in humidified atmosphere containing 5 % CO_2_. All the experiments were performed in RPMI-1640 containing 5 % fetal bovine serum.

### Cell growth studies by 3-(4,5-dimethylthiazol-2-yl)-2,5-diphenyltetrazolium bromide (MTT) assays

10^4^ cells were incubated in triplicate in a 96-well plate in a final volume of 0.2 ml for 48 h at 37 °C. Cell viability assay using MTT was performed as described previously [57].

### Live dead assay

To determine cell death, Live-Dead assay (Invitrogen, Eugene, OR) was used as described by the manufacturer. HT29 and Caco-2 cells were treated either alone with NS398, and Thiostrepton or in combination as described in the legends. Following incubation for 48 h, cells were suspended in 1 ml PBS containing 50 mM calcein AM and 8 mM ethidium homodimer and cells were incubated in the dark for 20 min. 50 μl of suspension was transferred on slides and visualized under an Olympus fluorescent microscope using a longpass filter.

### Soft agar colony assay

Soft agar colony experiments were performed according to the manufacturer’s protocol (Cheminon International, Temecula, CA, USA). Briefly, after treatment of cells with NS398, Thiostrepton or a combination of the two inhibitors for 48 h, 2500 cells were plated in 0.5 ml culture medium containing 0.4 % (v/v) top agar and 20 % fetal bovine serum (FBS) layered over a basal layer of 0.8 % (v/v) agar and 20 % FBS with culture medium and allowed to grow for 4 weeks as described previously (our REF). Following 4 weeks incubation, cells were stained at a final concentration of 1 mg/ml cell stain solution that was supplied with the kit.

### Cell invasion and migration assay

Cell invasion and migration assay were performed using 24-well Transwell Permeable Supports with 8-lM pores (Corning, Lowell, MA). Briefly, after treatment of cells with NS398, Thiostrepton or a combination of the two inhibitors for 48 h, cells were harvested, counted again and 1.25 x 105 cells were suspended in serum-free medium and seeded into Transwell inserts either uncoated (for migration assay) or coated (for invasion assay) with growth factor-reduced Matrigel (BD Biosciences, Bedford, MA). Bottom wells were filled with complete media for 24 h. After incubation of 24 h, filters containing the cells were stained with Diff-Quick stain set (Fisher Scientific, Pittsburg, PA), photographed under a fluorescent microscope and manual cell counts were obtained [41].

### Gene Silencing using siRNA

FoxM1 siRNA, Cox-2 siRNA and Scrambled control siRNA were purchased from Qiagen (Valencia, CA, USA). Cells were transfected using Lipofectamine 2000 (Invitrogen, Carlsbad, CA) for 6 h following which the lipid and siRNA complex was removed and fresh growth medium was added. Cells were lysed 48 h after transfection and specific protein levels were determined by Western Blot analysis with specific antibodies.

### Annexin V/PI dual staining

HT29 and Caco-2 cells were treated either with NS398, Thiostrepton or in combination as described in the legends. For detection of apoptosis, cells were harvested and percentage apoptosis was measured by flow cytometry after staining with flourescein-conjugated annexin-V and propidium iodide (PI) (Molecular probes, Eugene, OR) [58].

### Measurement of mitochondrial membrane potential

Cells were treated with NS398 and Thiostrepton as described in the legends for 48 h, washed twice with PBS, and re-suspended in mitochondrial incubation buffer. JC1 staining and flow cytometry were done as described previously.

### Cell lysis and immunoblotting

Cells were lysed as previously described [44]. Proteins were immunoblotted with different antibodies and visualized by the enhanced chemiluminescence (Amersham, Piscataway, NJ) method.

### Detection of Bax conformational changes

Detection of Bax conformation was performed as previously described [44]. In brief, HT29 cells were treated with combination of 10 μM NS398 and 5 μM Thiostrepton for indicated time periods after which proteins were lysed and immunoblotted using N20 Bax polyclonal antibody.

### Animals and xenograft study

Six weeks old nude mice were obtained from Jackson Laboratories (Maine, USA) and maintained in a pathogen free animal facility at least 1 week before use. All animal studies were done in accordance with institutional guidelines. For Xenograft study, mice were inoculated sub-cutaneously into the right abdominal quadrant with 10x10^6^ cells of HT29 in 200 μL PBS. After 1 week, mice were randomly assigned into four groups: The first group received DMSO. The three groups received N398 (15 mg/kg), Thiostrepton (150 mg/kg) and combination of 15 mg/kg NS398 and 150 mg/kg Thiostrepton, intra-peritoneally respectively. Mice were given these drugs twice weekly. The body weight and tumor volume of each mouse was monitored weekly. The tumor volume was measured as described previously [31]. After 5 weeks treatment, mice were sacrificed and individual tumors were weighed, then snap-frozen in liquid nitrogen for storage.

## Additional files

Additional file 1: Figure S1. Kaplan-Meier survival curve of CRC cases showing co-expression of Cox-2 and FoxM1 as compared to cases with normal or reduced expression (*p* = 0.4796). Additional file 2: Figure S2. DLD1 cells were incubated with 0-100 μM NS398 for 48 h. Cell viability was measured by MTT assays as described in Materials and Methods. The graph displays the mean +/- SD (standard deviation) of three independent experiments, **p* < 0.05, statistically significant (Students *t*-test). Additional file 3: Figure S3. LOVO cells were incubated with 0-100 μM NS398, 0-25 μM Thiostrepton or a combination of both drugs for 48 h. Cell viability was measured by MTT assays as described in Materials and Methods. The graph displays the mean +/- SD (standard deviation) of three independent experiments, **p* < 0.05, statistically significant (Students *t*-test). Additional file 4: Table S1. Combination Index calculation using Chou and Talalay method in Caco-2 cell line. Additional file 5: Table S2. Combination Index calculation using Chou and Talalay method in HT29 cell line. Additional file 6: Figure S4. (A and B) Caco-2 (A) and HT29 (B) cells were incubated with either 10 μM NS398, 5 μM Thiostrepton or a combination of both drugs for indicated time points. Cell viability was measured by MTT assays as described in Materials and Methods. The graph displays the mean +/- SD (standard deviation) of three independent experiments, **p* < 0.05, statistically significant (Students *t*-test). Additional file 7: Table S3. Combination Index calculation using Chou and Talalay method in DLD1 cell line. Additional file 8: Table S4. Combination Index calculation using Chou and Talalay method in LOVO cell line. Additional file 9: Table S5. Details of primary antibodies used in the study.
