# Supplementary figures and images for: Co-targeting of Cyclooxygenase-2 and FoxM1 is a viable strategy in inducing anticancer effects in colorectal cancer cells
Source: Mol Cancer. 2015 Jul 10;14:131. doi: 10.1186/s12943-015-0406-1 (PMC4861127; doi:10.1186/s12943-015-0406-1)

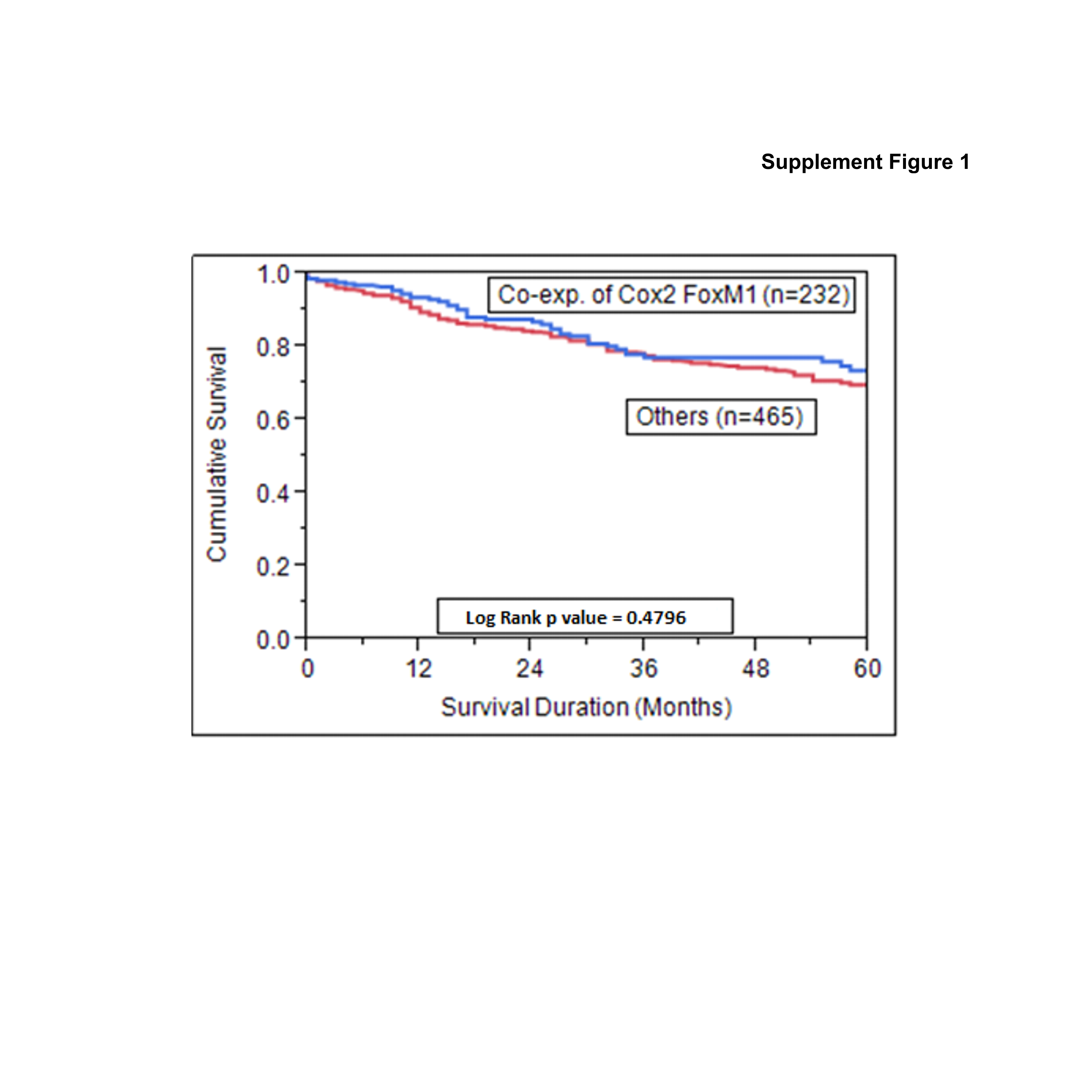

Supplement: Additional file 1: Figure S1. — Kaplan-Meier survival curve of CRC cases showing co-expression of Cox-2 and FoxM1 as compared to cases with normal or reduced expression (p = 0.4796). [file 12943_2015_406_MOESM1_ESM.tif]

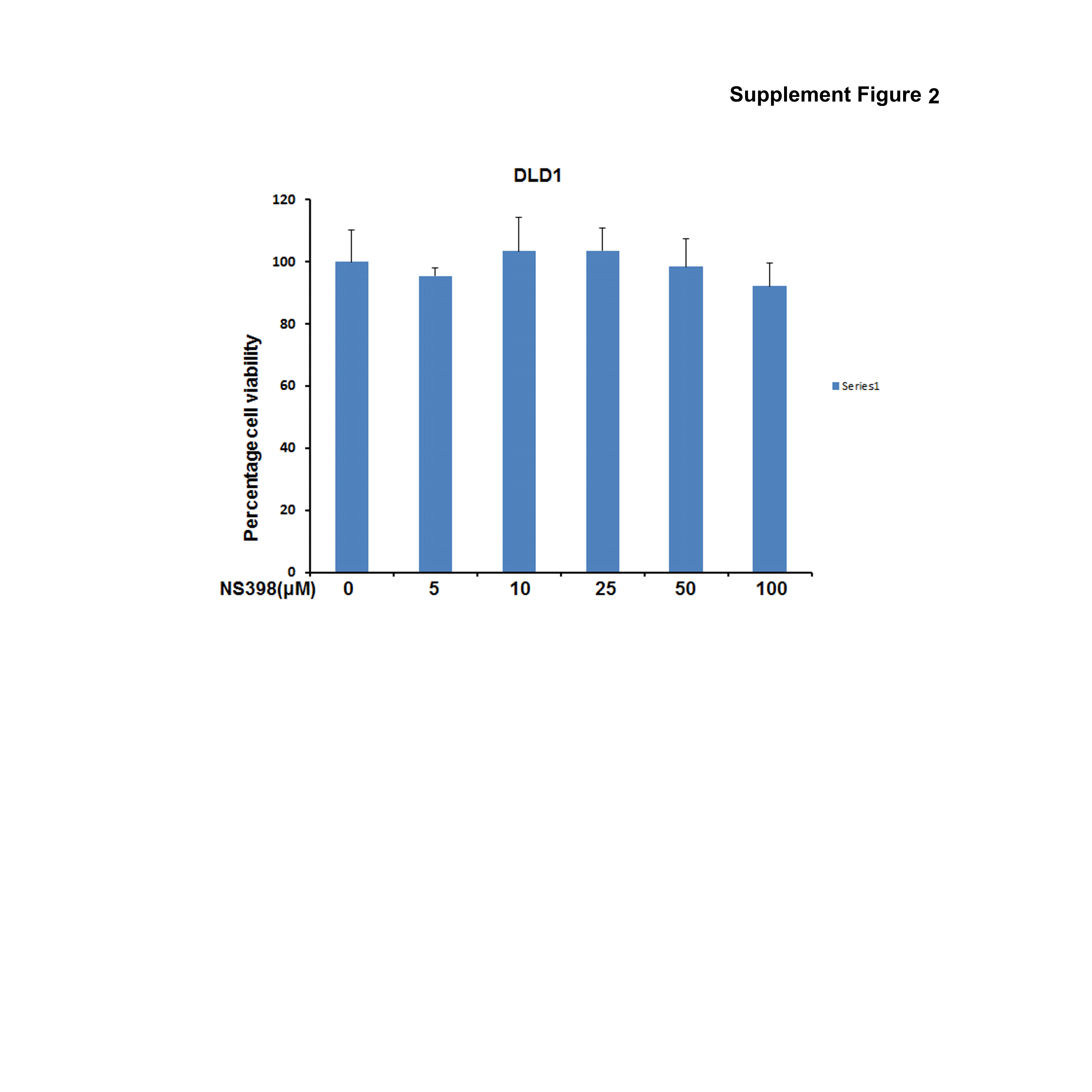

Supplement: Additional file 2: Figure S2. — DLD1 cells were incubated with 0-100 μM NS398 for 48 h. Cell viability was measured by MTT assays as described in Materials and Methods. The graph displays the mean +/- SD (standard deviation) of three independent experiments, *p < 0.05, statistically significant (Students t-test). [file 12943_2015_406_MOESM2_ESM.tif]

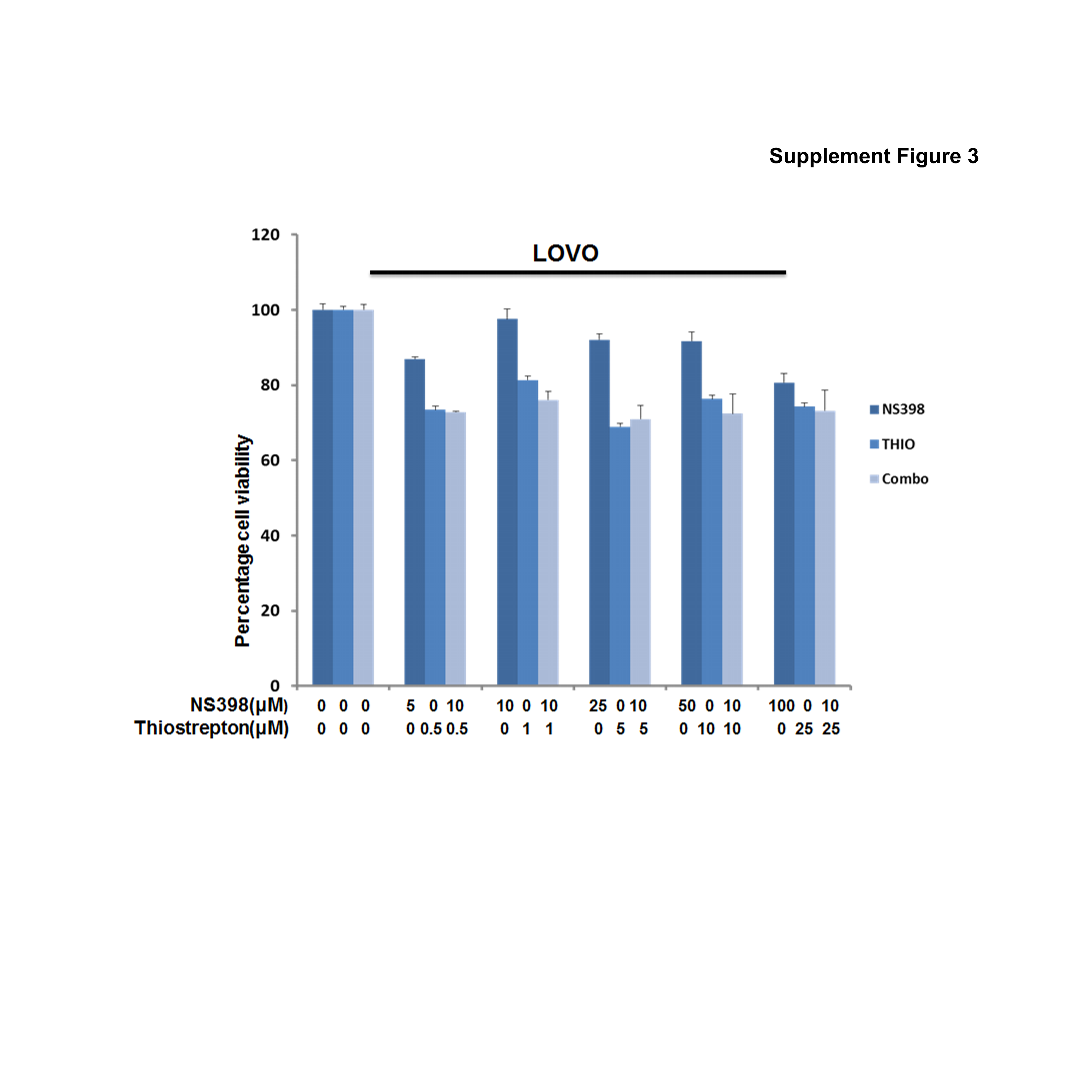

Supplement: Additional file 3: Figure S3. — LOVO cells were incubated with 0-100 μM NS398, 0-25 μM Thiostrepton or a combination of both drugs for 48 h. Cell viability was measured by MTT assays as described in Materials and Methods. The graph displays the mean +/- SD (standard deviation) of three independent experiments, *p < 0.05, statistically significant (Students t-test). [file 12943_2015_406_MOESM3_ESM.tif]

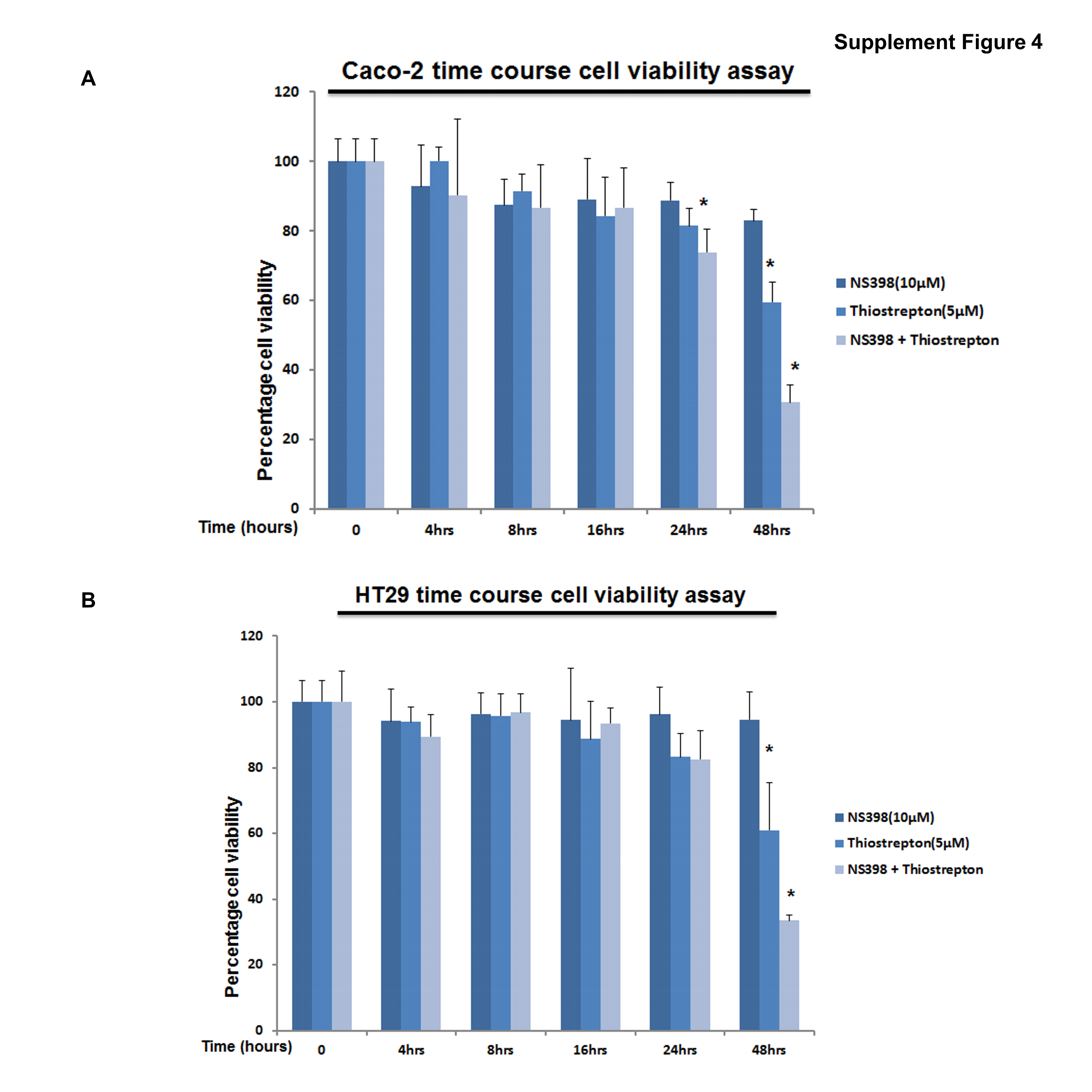

Supplement: Additional file 6: Figure S4. — (A and B) Caco-2 (A) and HT29 (B) cells were incubated with either 10 μM NS398, 5 μM Thiostrepton or a combination of both drugs for indicated time points. Cell viability was measured by MTT assays as described in Materials and Methods. The graph displays the mean +/- SD (standard deviation) of three independent experiments, *p < 0.05, statistically significant (Students t-test). [file 12943_2015_406_MOESM6_ESM.tif]
