# Supplementary material for: Co-targeting of Cyclooxygenase-2 and FoxM1 is a viable strategy in inducing anticancer effects in colorectal cancer cells
Source: Mol Cancer. 2015 Jul 10;14:131. doi: 10.1186/s12943-015-0406-1 (PMC4861127; doi:10.1186/s12943-015-0406-1)
Supplement: Additional file 4: Table S1. — Combination Index calculation using Chou and Talalay method in Caco-2 cell line. [file 12943_2015_406_MOESM4_ESM.doc]

**Supplement Table 1:** Combination Index calculation using Chou and Talalay method in CRC cell lines:

--------------------------------------------------------------------**Caco-2**--------------------------------------------------------------

| | Thiostrepton(µM) | NS398(µM) | Fractional effect (Fa) | Combination Index (CI) | Dose Reduction Index (DRI)  Thiostrepton (µM) | Dose Reduction Index (DRI)  NS398 (µM) | | --- | --- | --- | --- | --- | --- | | 0.5 |  | 0.017 |  |  |  | | 1.0 |  | 0.054 |  |  |  | | 5.0 |  | 0.353 |  |  |  | | 10 |  | 0.520 |  |  |  | | 25 |  | 0.71 |  |  |  | |
| --- | --- | --- | --- | --- | --- | --- | --- | --- | --- | --- | --- | --- | --- | --- | --- | --- | --- | --- | --- | --- | --- | --- | --- | --- | --- | --- | --- | --- | --- | --- | --- | --- | --- | --- | --- | --- |

**Median Dose (Dm) = 10.07µM**

**Exponent shape of curve (m) = 1.27079 ±0.083407**

**Linear correlation coefficient (r) = 0.99360**

| Thiostrepton(µM) | NS398(µM) | Fractional effect (Fa) | Combination Index (CI) | Dose Reduction Index (DRI)  Thiostrepton (µM) | Dose Reduction Index (DRI)  NS398 (µM) |
| --- | --- | --- | --- | --- | --- |
|  | 1 | 0.054 |  |  |  |
|  | 10 | 0.021 |  |  |  |
|  | 25 | 0.171 |  |  |  |
|  | 50 | 0.654 |  |  |  |
|  | 100 | 0.840 |  |  |  |

**Median Dose (Dm) = 53.61µM**

**Exponent shape of curve (m) = 1.02013 ±0.461628**

Linear correlation coefficient (r) = 0.78706

| Thiostrepton(µM) | NS398(µM) | Fractional effect (Fa) | Combination Index (CI) | Dose Reduction Index (DRI)  Thiostrepton (µM) | Dose Reduction Index (DRI)  NS398 (µM) |
| --- | --- | --- | --- | --- | --- |
| 0.5 | 10 | 0.060 | 3.20 | 2.31 | 0.361 |
| 1.0 | 10 | 0.212 | 0.955 | 3.584 | 1.480 |
| 5.0 | 10 | 0.703 | 0.332 | 3.968 | 12.47 |
| 10 | 10 | 0.724 | 0.537 | 2.151 | 13.80 |
| 25 | 10 | 0.793 | 0.913 | 1.159 | 20.00 |
