# Supplementary material for: Co-targeting of Cyclooxygenase-2 and FoxM1 is a viable strategy in inducing anticancer effects in colorectal cancer cells
Source: Mol Cancer. 2015 Jul 10;14:131. doi: 10.1186/s12943-015-0406-1 (PMC4861127; doi:10.1186/s12943-015-0406-1)
Supplement: Additional file 5: Table S2. — Combination Index calculation using Chou and Talalay method in HT29 cell line. [file 12943_2015_406_MOESM5_ESM.doc]

**Supplement Table 2:** Combination Index calculation using Chou and Talalay method in CRC cell lines:

--------------------------------------------------------------------**HT-29**--------------------------------------------------------------

| | Thiostrepton(µM) | NS398(µM) | Fractional effect (Fa) | Combination Index (CI) | Dose Reduction Index (DRI)  Thiostrepton (µM) | Dose Reduction Index (DRI)  NS398 (µM) | | --- | --- | --- | --- | --- | --- | | 0.5 |  | 0.010 |  |  |  | | 1.0 |  | 0.140 |  |  |  | | 5.0 |  | 0.313 |  |  |  | | 10 |  | 0.406 |  |  |  | | 25 |  | 0.706 |  |  |  | |
| --- | --- | --- | --- | --- | --- | --- | --- | --- | --- | --- | --- | --- | --- | --- | --- | --- | --- | --- | --- | --- | --- | --- | --- | --- | --- | --- | --- | --- | --- | --- | --- | --- | --- | --- | --- | --- |

**Median Dose (Dm) = 20.14µM**

**Exponent shape of curve (m) = 1.23136 ±0.248089**

**Linear correlation coefficient (r) = 0.94416**

| Thiostrepton(µM) | NS398(µM) | Fractional effect (Fa) | Combination Index (CI) | Dose Reduction Index (DRI)  Thiostrepton (µM) | Dose Reduction Index (DRI)  NS398 (µM) |
| --- | --- | --- | --- | --- | --- |
|  | 1 | 0.030 |  |  |  |
|  | 10 | 0.055 |  |  |  |
|  | 25 | 0.170 |  |  |  |
|  | 50 | 0.550 |  |  |  |
|  | 100 | 0.770 |  |  |  |

**Median Dose (Dm) = 59.6µM**

**Exponent shape of curve (m) = 1.01413 ±0.261802**

**Linear correlation coefficient (r) = 0.91290**

| Thiostrepton(µM) | NS398(µM) | Fractional effect (Fa) | Combination Index (CI) | Dose Reduction Index (DRI)  Thiostrepton (µM) | Dose Reduction Index (DRI)  NS398 (µM) |
| --- | --- | --- | --- | --- | --- |
| 0.5 | 10 | 0.060 | 2.987 | 2.17 | 0.396 |
| 1.0 | 10 | 0.118 | 1.723 | 1.98 | 0.821 |
| 5.0 | 10 | 0.627 | 0.424 | 3.09 | 9.96 |
| 10 | 10 | 0.838 | 0.293 | 3.85 | 30.17 |
| 25 | 10 | 0.936 | 0.291 | 3.58 | 84.07 |
