# Supplementary material for: Co-targeting of Cyclooxygenase-2 and FoxM1 is a viable strategy in inducing anticancer effects in colorectal cancer cells
Source: Mol Cancer. 2015 Jul 10;14:131. doi: 10.1186/s12943-015-0406-1 (PMC4861127; doi:10.1186/s12943-015-0406-1)
Supplement: Additional file 7: Table S3. — Combination Index calculation using Chou and Talalay method in DLD1 cell line. [file 12943_2015_406_MOESM7_ESM.doc]

**Supplement Table 3:** Combination Index calculation using Chou and Talalay method in CRC cell lines:

------------------------------------------------------------------- **DLD1** --------------------------------------------------------------

| | Thiostrepton(µM) | NS398(µM) | Fractional effect (Fa) | Combination Index (CI) | Dose Reduction Index (DRI)  Thiostrepton (µM) | Dose Reduction Index (DRI)  NS398 (µM) | | --- | --- | --- | --- | --- | --- | | 0.5 |  | 0.40 |  |  |  | | 1.0 |  | 0.52 |  |  |  | | 5.0 |  | 0.51 |  |  |  | | 10 |  | 0.63 |  |  |  | | 25 |  | 0.68 |  |  |  | |
| --- | --- | --- | --- | --- | --- | --- | --- | --- | --- | --- | --- | --- | --- | --- | --- | --- | --- | --- | --- | --- | --- | --- | --- | --- | --- | --- | --- | --- | --- | --- | --- | --- | --- | --- | --- | --- |

**Median Dose (Dm) = 1.61µM**

**Exponent shape of curve (m) = 0.25602 ±0.064799**

**Linear correlation coefficient (r) = 0.91586**

| Thiostrepton(µM) | NS398(µM) | Fractional effect (Fa) | Combination Index (CI) | Dose Reduction Index (DRI)  Thiostrepton (µM) | Dose Reduction Index (DRI)  NS398 (µM) |
| --- | --- | --- | --- | --- | --- |
|  | 1 | 0.05 |  |  |  |
|  | 10 | 0.06 |  |  |  |
|  | 25 | 0.05 |  |  |  |
|  | 50 | 0.08 |  |  |  |
|  | 100 | 0.089 |  |  |  |

**Median Dose (Dm) = 3.9 x10^9^µM**

**Exponent shape of curve (m) = 0.13601 ±0.055657**

**Linear correlation coefficient (r) = 0.81586**

| Thiostrepton(µM) | NS398(µM) | Fractional effect (Fa) | Combination Index (CI) | Dose Reduction Index (DRI)  Thiostrepton (µM) | Dose Reduction Index (DRI)  NS398 (µM) |
| --- | --- | --- | --- | --- | --- |
| 0.5 | 10 | 0.45 | 0.677 | 1.47 | 9x10^7^ |
| 1.0 | 10 | 0.50 | 0.618 | 3.595 | 1.73x10^8^ |
| 5.0 | 10 | 0.60 | 0.634 | 1.576 | 7.7x10^9^ |
| 10 | 10 | 0.67 | 0.389 | 2.571 | 7.21x10^10^ |
| 25 | 10 | 0.70 | 0.565 | 1.771 | 2.0x10^11^ |
