# Supplementary material for: Co-targeting of Cyclooxygenase-2 and FoxM1 is a viable strategy in inducing anticancer effects in colorectal cancer cells
Source: Mol Cancer. 2015 Jul 10;14:131. doi: 10.1186/s12943-015-0406-1 (PMC4861127; doi:10.1186/s12943-015-0406-1)
Supplement: Additional file 8: Table S4. — Combination Index calculation using Chou and Talalay method in LOVO cell line. [file 12943_2015_406_MOESM8_ESM.doc]

**Supplement Table 4:** Combination Index calculation using Chou and Talalay method in CRC cell lines:

------------------------------------------------------------------- **LOVO** --------------------------------------------------------------

| | Thiostrepton(µM) | NS398(µM) | Fractional effect (Fa) | Combination Index (CI) | Dose Reduction Index (DRI)  Thiostrepton (µM) | Dose Reduction Index (DRI)  NS398 (µM) | | --- | --- | --- | --- | --- | --- | | 0.5 |  | 0.256 |  |  |  | | 1.0 |  | 0.187 |  |  |  | | 5.0 |  | 0.212 |  |  |  | | 10 |  | 0.238 |  |  |  | | 25 |  | 0.258 |  |  |  | |
| --- | --- | --- | --- | --- | --- | --- | --- | --- | --- | --- | --- | --- | --- | --- | --- | --- | --- | --- | --- | --- | --- | --- | --- | --- | --- | --- | --- | --- | --- | --- | --- | --- | --- | --- | --- | --- |

**Median Dose (Dm) = 6.2 x 10^15^µM**

**Exponent shape of curve (m) = 0.03459 ± 0.059692**

**Linear correlation coefficient (r) = 0.31731**

| Thiostrepton(µM) | NS398(µM) | Fractional effect (Fa) | Combination Index (CI) | Dose Reduction Index (DRI)  Thiostrepton (µM) | Dose Reduction Index (DRI)  NS398 (µM) |
| --- | --- | --- | --- | --- | --- |
|  | 1 | 0.13 |  |  |  |
|  | 10 | 0.03 |  |  |  |
|  | 25 | 0.08 |  |  |  |
|  | 50 | 0.083 |  |  |  |
|  | 100 | 0.194 |  |  |  |

**Median Dose (Dm) = 4.9 x 10^8^µM**

**Exponent shape of curve (m) = 0.13072 ± 0.0281483**

**Linear correlation coefficient (r) = 0.25898**

| Thiostrepton(µM) | NS398(µM) | Fractional effect (Fa) | Combination Index (CI) | Dose Reduction Index (DRI)  Thiostrepton (µM) | Dose Reduction Index (DRI)  NS398 (µM) |
| --- | --- | --- | --- | --- | --- |
| 0.5 | 10 | 0.25 | 0.500 | 202.9 | 1.12x10^4^ |
| 1.0 | 10 | 0.17 | 12.8 | 7.1x10^2^ | 268.8 |
| 5.0 | 10 | 0.22 | 6.16 | 16.2 | 3.1x10^3^ |
| 10 | 10 | 0.22 | 12.32 | 8.1 | 3.1x10^3^ |
| 25 | 10 | 0.27 | 0.12 | 82 | 2.4x10^4^ |
