# Supplementary material for: Co-targeting of Cyclooxygenase-2 and FoxM1 is a viable strategy in inducing anticancer effects in colorectal cancer cells
Source: Mol Cancer. 2015 Jul 10;14:131. doi: 10.1186/s12943-015-0406-1 (PMC4861127; doi:10.1186/s12943-015-0406-1)
Supplement: Additional file 9: Table S5. — Details of primary antibodies used in the study. [file 12943_2015_406_MOESM9_ESM.docx]

**Supplementary Table 5**: Details of primary antibodies used in the study.

| **Antibody** | **Cut-off** | **Incidence** | **Clone** | **Company** | **Dilution** | **Retrieval** | **Detection** |
| --- | --- | --- | --- | --- | --- | --- | --- |
| Cox-2 | H-score ≥170 | 60.6% | polyclonal | Abcam | 1:1000 | pH6,pressure cooker | Envision+ |
| FoxM1(K-19) | H-score ≥50 | 50.3% | polyclonal | SantaCruz BT | 1:2500 | pH9,pressure cooker | Envision+ |
| p-AKT (Ser473) | Int-score 2+/3+ | 72.3% | 736E11 | Cell Signalling | 1:20 | pH9,pressure cooker | Envision+ |
| Ki-67 | ≥50% nuclear | 87.7% | MIB-1 | DAKO | 1:500 | pH9, pressure cooker | Envision+ |
| MMP-9 | H-score ≥130 | 51.4% | 56-2A4 | Calbiochem | 1:2000 | pH6,pressure cooker | Envision+ |
